# Functional Analysis of a Salicylate Hydroxylase in *Sclerotinia sclerotiorum*

**DOI:** 10.3390/jof9121169

**Published:** 2023-12-05

**Authors:** Shengfei He, Kun Huang, Baoge Li, Guodong Lu, Airong Wang

**Affiliations:** 1 State Key Laboratory of Ecological Pest Control for Fujian and Taiwan Crops, Fujian Agriculture and Forestry University, Fuzhou 350002, China; hsf70880@163.com (S.H.); kunhuang0126@163.com (K.H.); lbg1212@163.com (B.L.); lgd@fafu.edu.cn (G.L.); 2 Haixia Institute of Science and Technology, Fujian Agriculture and Forestry University, Fuzhou 350002, China

**Keywords:** *Sclerotinia sclerotiorum*, salicylate hydroxylase, salicylic acid, virulence

## Abstract

Salicylic acid plays a crucial role during plant defense to *Sclerotinia sclerotiorum*. Some bacteria and a few fungi can produce salicylate hydroxylase to degrade SA to suppress plant defense and increase their virulence. But there has been no single salicylate hydroxylase in *Sclerotinia sclerotiorum* identified until now. In this study, we found that *SS1G_02963* (*SsShy1*), among several predicted salicylate hydroxylases in *S. sclerotiorum*, was induced approximately 17.6-fold during infection, suggesting its potential role in virulence. SsShy1 could catalyze the conversion of SA to catechol when heterologous expression in *E. coli*. Moreover, overexpression of *SsShy1* in *Arabidopsis thaliana* decreased the SA concentration and the resistance to *S. sclerotiorum*, confirming that SsShy1 is a salicylate hydroxylase. Deletion mutants of *SsShy1* (∆*Ssshy1*) showed slower growth, less sclerotia production, more sensitivity to exogenous SA, and lower virulence to *Brassica napus*. The complemented strain with a functional *SsShy1* gene recovered the wild-type phenotype. These results indicate that SsShy1 plays an important role in growth and sclerotia production of *S. sclerotiorum*, as well as the ability to metabolize SA affects the virulence of *S. sclerotiorum*.

## 1. Introduction

A plant’s defense response is triggered and activated when a pathogen invades a plant. The induced defenses in plants can be divided into two types: pathogen-associated molecular patterns (PAMP)-triggered immunity (PTI) and effector-triggered immunity (ETI) [1]. Salicylic acid (SA), also known as 2-hydroxybenzoic acid [2], plays a significant role in both PTI and ETI immune responses [1,3,4], amplifying basic defense and local immune responses, and establishing systemic-acquired resistance (SAR) [5,6,7]. Evidence that SA is a key defense-signaling hormone comes from studies of SA-deficient tobacco and Arabidopsis [8,9]. PTI, ETI, and SAR were inhibited by the expression of NahG encoding the SA-metabolizing enzyme salicylate hydroxylase from *Pseudomonas putida* [8,9]. Further analysis revealed that mutations in endogenous components associated with pathogen-induced accumulation of SA also lead to reduced immunity [10,11,12]. In many plants, SA accumulation and downstream signaling play a central role in plant defense against invasive pathogens [7,13].

Salicylate hydroxylase is a flavin protein monooxygenase and a central component of the naphthalene degradation pathway [14]. This enzyme binds SA and nicotinamide adenine dinucleotide (NADH) or nicotinamide adenine dinucleotide phosphate (NADPH) to form an enzyme–substrate complex. Molecular oxygen then binds to this complex, producing catechol, CO_2_, and H_2_O [15]. Research has shown that genes encoding salicylate hydroxylase exist in many bacteria, including multiple members of *Pseudomonas*, *Ralstonia*, *Bacillus*, *Agrobacterium*, and other genera. For instance, *Ralstonia solanacearum* can degrade plant SA through salicylate hydroxylase, reducing SA inhibition on itself and increasing its pathogenicity on host plants [16]. Salicylate hydroxylase from the Asian species of *Candidatus* Liberibacter asiaticus can also degrade plant SA and suppress plant defense response, thereby increasing the susceptibility of citrus to both pathogenic and non-pathogenic citrus *Xanthoomonas* [17]. The expression of the bacterial salicylate hydroxylase encoding gene *NahG* in tobacco and *Arabidopsis thaliana* transforms SA into catechol, blocking SA accumulation, and preventing the plant from inducing SAR [8,9]. This highlights the importance of salicylate hydroxylase. Salicylate hydroxylase in fungi has not been extensively studied. For example, Shy1, a functional salicylate hydroxylase, was identified in *U. maydis* and is required for growth on plates with SA as the sole carbon source; however, its inactivation had no significant effect on virulence [18]. The deletion of FgShy1 encoding a salicylate hydroxylase in *Fusarium graminearum* seriously affected the degradation of SA but had no impact on the pathogenicity of *Fusarium head blight* (FHB) [19,20]. Another salicylate hydroxylase, FgNahG in *F. graminearum*, was identified. Lowering the SA concentration in plants and disrupting the *FgNahG* gene inhibited the development of head blight symptoms [21]. The salicylate hydroxylase FoSAH1 in *Fusarium oxysporum* significantly attenuated the toxicity of exogenous SA and transgenic plants, with FoSAH1 RNA interference significantly preventing the occurrence of *Fusarium wilt* [22].

*Sclerotinia sclerotiorum* is a global pathogen that infects a wide range of host species and causes significant damage to economically important crops such as oil crops and vegetables [23]. Due to its extensive host range, research on this pathogen is still limited, and its pathogenic mechanisms are not yet well understood. Current agricultural control methods primarily rely on chemical control; however, the long-term and large-scale use of pesticides has led to the development of resistance in field strains. As a result, it is necessary to further investigate the pathogenic mechanisms of *S. sclerotiorum* to develop new and effective control strategies. Previous studied have shown that SA plays an important role in plant defense against *S. sclerotiorum* [24,25,26,27]. It is not yet clear whether salicylate hydroxylases in *S. sclerotiorum* are involved in the interaction with host plants. In this study, we have identified a salicylate hydroxylase in *S. sclerotiorum* and have further investigated its role in the pathogenesis of the fungus. A deeper understanding of the function of this salicylate hydroxylase in *S. sclerotiorum* could shed light on the pathogen’s interaction with host plants and contribute to the development of more targeted and effective control measures.

## 2. Materials and Methods

### 2.1. Fungal Strains, Plant Materials, and Culture Conditions

The wild-type strain of *S. sclerotiorum* 1980 was used as the wild-type control in this study. Deletion mutants and complemented strain were cultured at 22 °C in the dark on potato dextrose agar (PDA) medium containing 100 μg/mL hygromycin B or 100 μg/mL G418. The *Brassica napus* plants were grown in a growth chamber at 22 °C with a 16 h light cycle and a relative humidity of 70%.

### 2.2. Bioinformatic Analysis

For the identification of candidate members of salicylate hydroxylase in *S. sclerotiorum*, we used the sequences of FgShy1 and FgNahG in *F. graminearum* as references and performed a similarity search against the *S. sclerotiorum* proteome using the BLASTp program (http://fungi.ensembl.org/Sclerotinia_sclerotiorum/Tools/Blast?tl=ACwUdhH5J4suahdU-20550044-1806510280, accessed on 20 October 2022). Subsequently, all potential salicylate hydroxylases were validated using CDD (http://www.ncbi.nlm.nih.gov/Structure/cdd/wrpsb.cgi, accessed on 20 October 2022), Pfam (http://pfam.xfam.org/, accessed on 20 October 2022), and SMART (http://smart.emblheidelberg.de/, accessed on 20 October 2022) analyses. Lastly, a phylogenetic tree was constructed using MEGA 10.0.5 based on the maximum likelihood method, with bootstrapping carried out 1000 times.

### 2.3. Expression Vector Construction and Salicylate Hydroxylase Activity Assay

The cDNA sequences of selected candidate genes: *SS1G_02963*, *SS1G_12062*, *SS1G_12382*, *SS1G_04729*, and *FgShy1*, were amplified by PCR with the designated primer pairs listed in Table S1 with the name containing GST and then integrated into the pGEX-4T-1 vector. The recombinant vector was subsequently transformed into *Escherichia coli* BL21 (DE3) cells (Tiangen, Beijing, China) and screened on Luria–Bertani (LB) medium containing 100 µg/mL ampicillin. The positive clones were confirmed by PCR with primer pairs pGEX5’/pGEX3’ and sequenced. The proteins expressed by the positive clones were verified by electrophoresis in SDS-PAGE gel and staining by Coomassie brilliant blue (CBB) or Western blotting (Figure S1). Strains derived from mature liquid cultures were streaked onto LB plates with 0.1 mM IPTG and 1 mM SA. The strain expressing FgShy1 was employed as a positive control. The plates were incubated at 25 °C and observed for a color change to brown, indicating the conversion of SA to catechol and subsequent oxidation.

### 2.4. RNA Extraction and RT-PCR Analysis

Fungal mycelia and plant tissues infected with *S. sclerotiorum* were collected and ground into a powder in liquid nitrogen. Total RNA was isolated using the TianGen RNAprep pure Plant kit (Tiangen, Beijing, China). The first strand cDNA was synthesized from 1 µg RNA using the PrimeScript^TM^ RT reagent kit (Takara, Beijing, China) and subsequently utilized as a template for reverse transcription quantitative polymerase chain reaction (RT-qPCR). The primers are listed in Table S1 with the name containing “q”. *Tubulin* was used as an internal control for analysis of fungal genes expression and *ACTIN 2 (AtACT2)* was used as an internal control for analysis of genes expression in transgenic *A. thaliana* plants.

### 2.5. Mutant Generation and Complementation

The genomic DNA of *S. sclerotiorum* was extracted using a standard protocol with a cetyltrimethylammonium bromide (CTAB)-based buffer. The strategy for gene deletion in *S. sclerotiorum* was rooted in homologous recombination, which replaced the target gene with antibiotic resistance fragment to facilitate the screening of mutants. More specifically, the upstream and downstream flanking sequences of *SsShy1* were amplified using the primer pairs AF/AR and BF/BR, respectively. These sequences were then fused with the HY and YG divisions of the *HPH* gene via Splicing Overlap Extension (SOE)-PCR (Figure S1A). Protoplast preparation and PEG-mediated transformation of *S. sclerotiorum* were executed following Rollins’ protocol [28]. After screening on PDA medium containing 100 µg/mL hygromycin B, the transformants were verified by PCR and further confirmed by RT-PCR using primer pairs OF/OR and UA/H853.

To complement the gene function, the predicted native promoter of *SsShy1* was amplified in conjunction with the ORF full-length sequence with primers CF/CR and cloned into the pNAH-ONG plasmid. This recombinant plasmid was verified through sequencing and then introduced into the protoplasts of Δ*Ssshy1* by PEG-mediated transformation. The positive transformants were further confirmed by RT-PCR using primer pairs OF/OR and UA/H853.

### 2.6. Hypha Growth, Cell Wall Integrity, Oxalic Acid Production, and Exogenous SA Sensitivity Assay

All strains were cultured on PDA medium at 22 °C, which included either NaCl (0.6 M), Congo Red (CR, 0.2 mg/mL), bromophenol (50 μg/mL), or SA at concentrations of 0.3 mM, 1 mM, or 2.5 mM. Colony diameters were measured at 2 days post inoculation (dpi). The formation and quantification of sclerotia were assessed at 7 and 14 dpi. All these experiments were repeated five times.

### 2.7. Pathogenicity Assay

Two-day-old mycelial plugs were inoculated on detached leaves of *B. napus*. The inoculated leaflets were incubated at 22 °C in a humid chamber. Symptoms were observed and recorded at 36 h post-inoculation (hpi). All tests were repeated three times.

### 2.8. Transgenic Arabidopsis Construction and Quantification of SA in A. thaliana Leaves

The CDS of *SsShy1* was amplified with primers 104F/104R and cloned into the pEG104 plasmid. Post sequencing to verify the recombinant plasmid, it was transformed into *A. thaliana*. After being screened by spraying BASTA when the plants were 2–3 weeks old, the positive transgenic plants were confirmed by PCR with the primer pairs BarF/BarR, and further confirmed by RT-PCR with primer pairs *SS1G_02963* qF/qR; *AtACT2* was used as the internal control. The quantification of SA was carried out following the method described by Siciliano et al. [29].

### 2.9. Statistical Analysis

All graphs were generated using GraphPad Prism 9.0.0 software. Statistical significance was determined by *t*-test using GraphPad Prism 9.0.0 software.

## 3. Results

### 3.1. Identification of Genes Encoding Salicylate Hydroxylase in S. sclerotiorum

**Figure 1:**
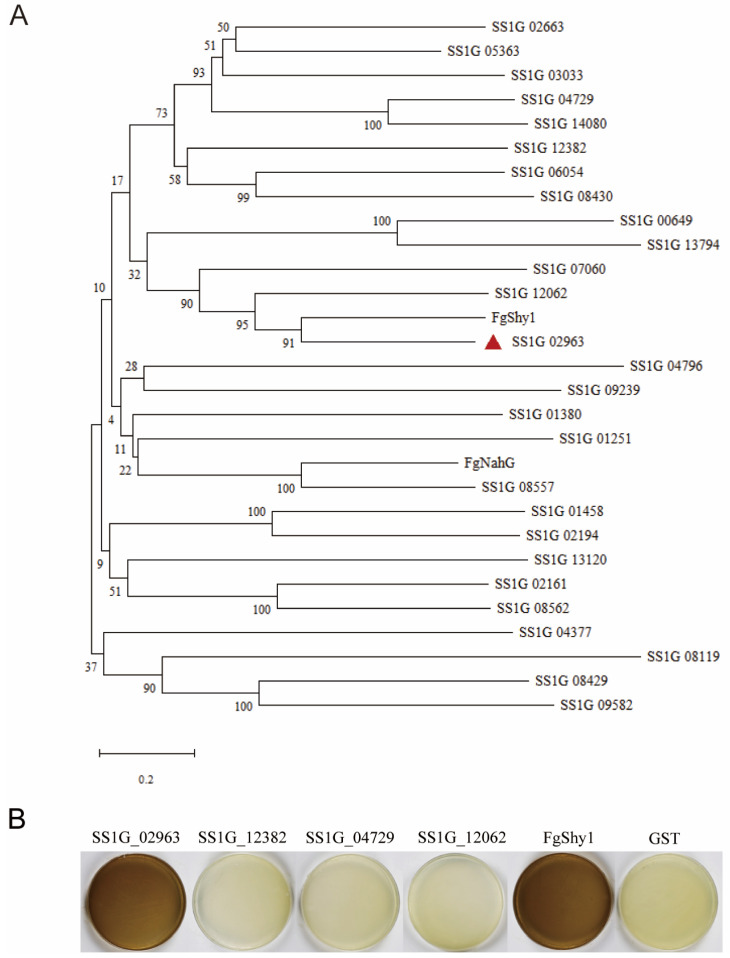
Identification of salicylate hydroxylase in *S. sclerotiorum*. (**A**) phylogenetic analysis of candidate salicylate hydroxylases in *S. sclerotiorum* with bootstrap 1000 times. (**B**) Colorimetric plate assay of enzyme activity. Positive control was the strain expressing FgShy1 and negative control was the strains expressing GST. The red triangle indicates the location of SsShy1.

Using salicylate hydroxylase Shy1 [19] and NahG [21] in *F. graminearum* to BLAST against the *S. sclerotiorum* database (https://fungi.ensembl.org/Sclerotinia_sclerotiorum/Info/Index, accessed on 20 October 2022), we identified 10 genes encoding salicylate hydroxylase. Phylogenetic analysis segregated these genes into three clades. Some proteins shared high homology within the FgShy1 clade, some resided in the FgNahG clade, and the remaining proteins were in the third clade (Figure 1A). We selected four proteins from the FgShy1 clade to analyze their activity, with two proteins (SS1G_02963 and SS1G_12062) closely related to FgShy1 and two proteins (SS1G_12382 and SS1G_04729) a little far away from FgShy1.

Since salicylate hydroxylase can convert SA into the brown substance catechol, we expressed SS1G_02963, SS1G_12062, SS1G_12382, and SS1G_04729 in *E. coli*. After confirmation by electrophoresis in SDS-PAGE gel, and staining by Coomassie brilliant blue (CBB) (Figure S1A) or western blotting (Figure S1B), the expression strains were cultured on LB medium containing 0.1 mM IPTG and 1 mM SA. The strains expressing FgShy1 were used as a positive control. Both the strain expressing SS1G_02963 and the positive control strain caused discoloration on the plates (Figure 1B), indicating that SA was converted to catechol. However, the strains expressing SS1G_12062, SS1G_12382, and SS1G_04729 were similar to the negative control, suggesting that amongst the selected four proteins, only SS1G_02963 had salicylate hydroxylase activity. Hence, we named it as SsShy1.

### 3.2. Overexpression of SsShy1 in Plant Reduced the Content of Plant SA and Increased Susceptibility to S. sclerotiorum

**Figure 2:**
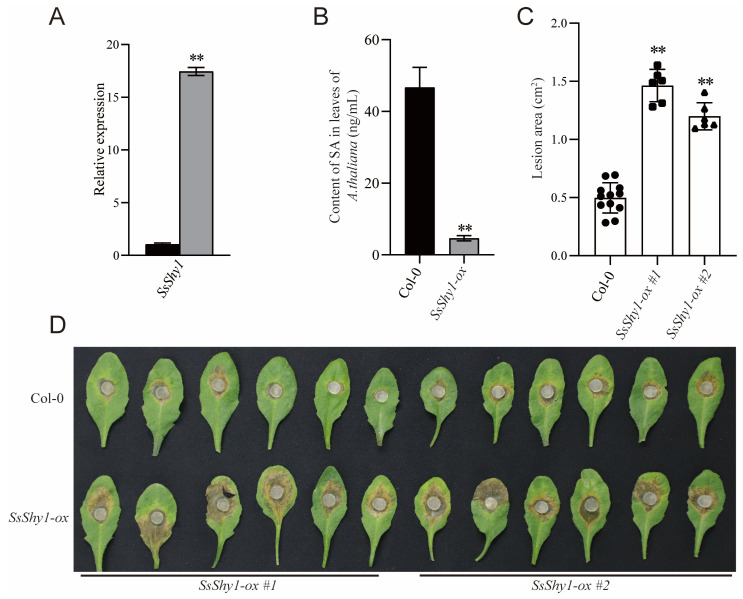
Evaluation of the function of SsShy1 in *A. thaliana*. (**A**) Expression levels of *SsShy1* at 24 h after infection into *B. napus*. *Tubulin* was used as an internal control. (**B**) SA contents in the leaves of the wild-type Col-0 and *SsShy1* transgenic plants at 25 days after germination. The error bars represent SD from three independent experiments. Asterisks represent significant differences (**, *p* < 0.01 by *t*-test). (**C**) Lesion sizes of the wild-type Col-0 and two independent lines of *SsShy1* transgenic plants infected by the wild-type strain of *S. sclerotiorum*. The error bars represent SD from replicates of one experiment. The black circles/squares/triangles in the column present replicates in one experiment. Asterisks represent significant differences (**, *p* < 0.01 by *t*-test). The experiment was repeated three times and the results were similar. (**D**) Symptoms in the leaves of the wild-type Col-0 and two independent lines of *SsShy1* transgenic plants at 24 h after inoculation with *S. sclerotiorum*. *SsShy1-ox* #1 and *SsShy1-ox* #2 were two independent lines of *SsShy1* transgenic *A. thaliana*.

To further investigate SsShy1’s activity, we first examined the expression level during the vegetative growth stage and host infection stage. We cultured the wild-type strain 1980 on PDA medium for 36 h, collected mycelia, and extracted total RNA. Simultaneously, we inoculated mycelial plugs on *B. napus* leaves for 24 h, then excised the lesion area and extracted total RNA. The RT-qPCR results demonstrate that the expression level of *SsShy1* was particularly significantly upregulated during the infection stage compared to the vegetative growth stage, up to approximately 17.6 times (Figure 2A). These findings suggest that *SsShy1* may play a role in the pathogenesis of *S. sclerotiorum*.

Then, we overexpressed the gene in *A. thaliana*. The positive transgenic lines were confirmed by PCR (Figure S2A). After confirming that the gene expression was normal by RT-PCR (Figure S2B), the SA content and susceptibility of the transgenic plants to *S. sclerotiorum* were analyzed. We found that the SA contents of transgenic *A. thaliana* were significantly lower than those of the wild type (Figure 2B). Upon inoculation with *S. sclerotiorum*, the lesions in the transgenic *A. thaliana* lines *SsShy1-ox* #1 and *SsShy1-ox* #2 were notably larger than those of the wild-type Col-0 plants at 24 hpi (Figure 2C,D). These results suggest that SsShy1 effectively degrades SA and can increase the virulence of *S. sclerotiorum*.

### 3.3. Deletion of SsShy1 Affected the Hyphae Growth and Sclerotia Production

To study the biological function of SsShy1 in *S. sclerotiorum*, we created a *SsShy1*-deleted mutant (Δ*Ssshy1*) using homologous recombination (Figure S3A). After confirmation by RT-PCR, the complemented strain (Δ*Ssshy1*/*SsShy1*) was constructed with the full-length sequence of *SsShy1* and further identified by RT-PCR (Figure S3B).

**Figure 3:**
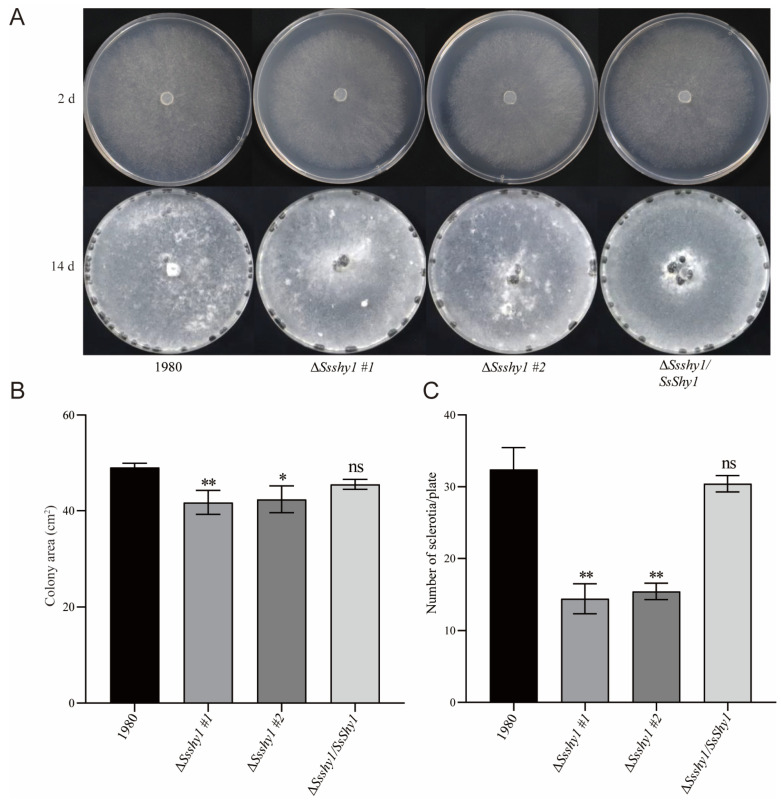
Deletion of *SsShy1* affected hyphae growth and sclerotia production. (**A**) Hyphae growth and sclerotia production in PDA at different days. (**B**) Statistical analysis of growth rate after incubated for 36 h at 22 °C in the dark. (**C**) Statistical analysis of the number of sclerotia produced at 14 dpi. The error bars are the standard deviations (SDs) from three independent experiments and asterisks represent significant difference between the wild type and mutants (ns, no significant difference; *, *p* < 0.05; **, *p* < 0.01 by *t*-test).

To ascertain SsShy1′s role in hyphae growth, we cultured Δ*Ssshy1*, Δ*Ssshy1*/*SsShy1*, and the wild-type strain (1980) on PDA medium for 36 h. There was no significant difference in colony morphology between the Δ*Ssshy1* mutant and the wild-type or Δ*Ssshy1*/*SsShy1*. However, the growth of the mutant Δ*Ssshy1* was noticeably lesser than that of the wild-type, while the complemented strain exhibited a similar mycelial growth rate as the wild-type (Figure 3A,B). These findings suggest that while *SsShy1* deletion did not influence the colony morphology, it did affect hyphae growth.

To establish whether SsShy1 regulated the development of sclerotia, Δ*Ssshy1*, Δ*Ssshy1/SsShy1*, and the wild-type strain were cultivated on PDA medium for 14 days, and the quantity of sclerotia produced was recorded, respectively, at 14 dpi. The number of sclerotia formed by Δ*Ssshy1/SsShy1* was comparable to that of the wild type, while the mutant Δ*Ssshy1* generated significantly fewer sclerotia than the wild type (Figure 3C). This indicates that SsShy1 contributes to the control of sclerotia formation and development.

### 3.4. SsShy1 Was Related to Cell Wall Integrity but Not OA Secretion

**Figure 4:**
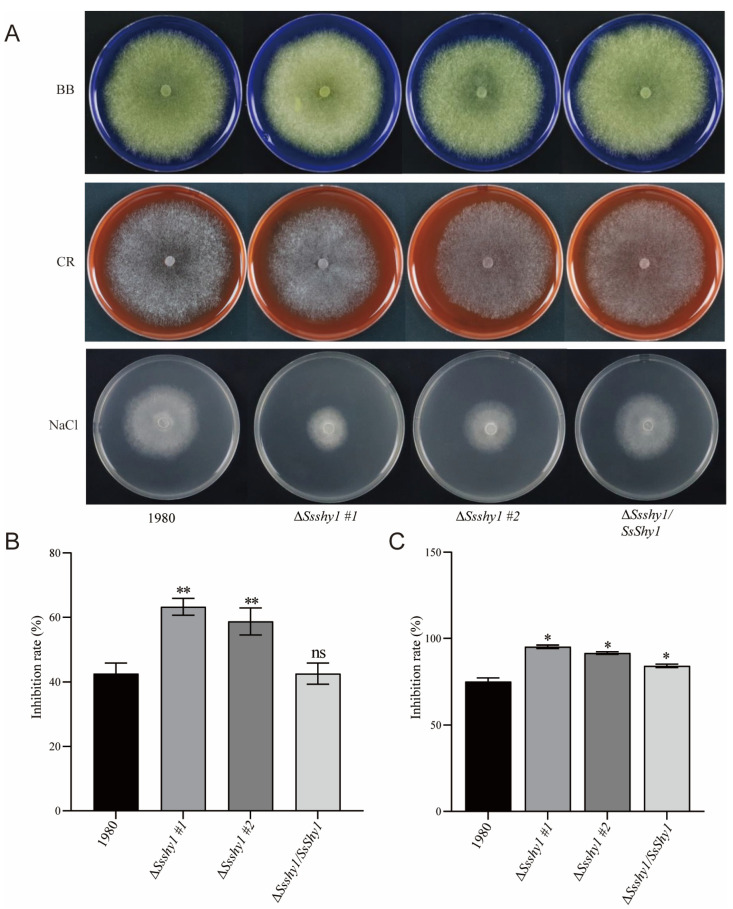
Deletion of *SsShy* did not affect OA accumulation but became more sensitivity to cell wall inhibitors. (**A**) The growth on different conditions. (**B**) Growth inhibition rates on PDA medium containing Congo red. (**C**) Growth inhibition rates on PDA medium containing 0.6 M NaCl. The error bars are SDs from three independent experiments and asterisks represent significant difference between the wild type and mutants (ns, no significant difference; *, *p* < 0.05; **, *p* < 0.01 by *t*-test).

Studies have demonstrated that oxalic acid (OA) is a crucial pathogenic factor produced by *S. sclerotinia* [30]. To investigate whether the deletion of *SsShy1* impacted OA production, we cultured the Δ*Ssshy1*, Δ*Ssshy1*/*SsShy1*, and wild-type strains on PDA medium containing 50 μg/mL bromophenol blue, and monitored the color changes of the medium after 36 h. As the colonies grew, the color of the medium changed from blue to yellow, indicating that the mycelia generated a significant amount of acidic substances, mainly OA, during the growth process. However, there was no marked difference in acid production between the Δ*Ssshy1* mutant and wild-type strains or Δ*Ssshy1*/*SsShy1* (Figure 4A), suggesting that the deletion of *SsShy1* did not affect the OA production.

The fungal cell wall is important in maintaining cell morphology and adaptability to the environment. We found that protoplasts were rapidly released from the mycelia of the Δ*Ssshy1* mutant during protoplasm preparation, suggesting an incomplete cell wall development. All strains were cultured in PDA medium containing various cell wall inhibitors for 2 days, after which their diameters were measured to calculate inhibition rates. The results showed that the growth of the complemented strain was similar to that of the wild-type strain, whereas Δ*Ssshy1* mutant growth was significantly inhibited in both 200 μg/mL Congo red (CR) (Figure 4A,B) and 0.6 M NaCl treatments (Figure 4A,C), indicating that the cell wall of the Δ*Ssshy1* mutant lacked integrity. This finding suggests that SsShy1 is involved in the organization of the cell wall.

### 3.5. Deletion of SsShy1 Increased Sensitivity to Exogenous SA

**Figure 5:**
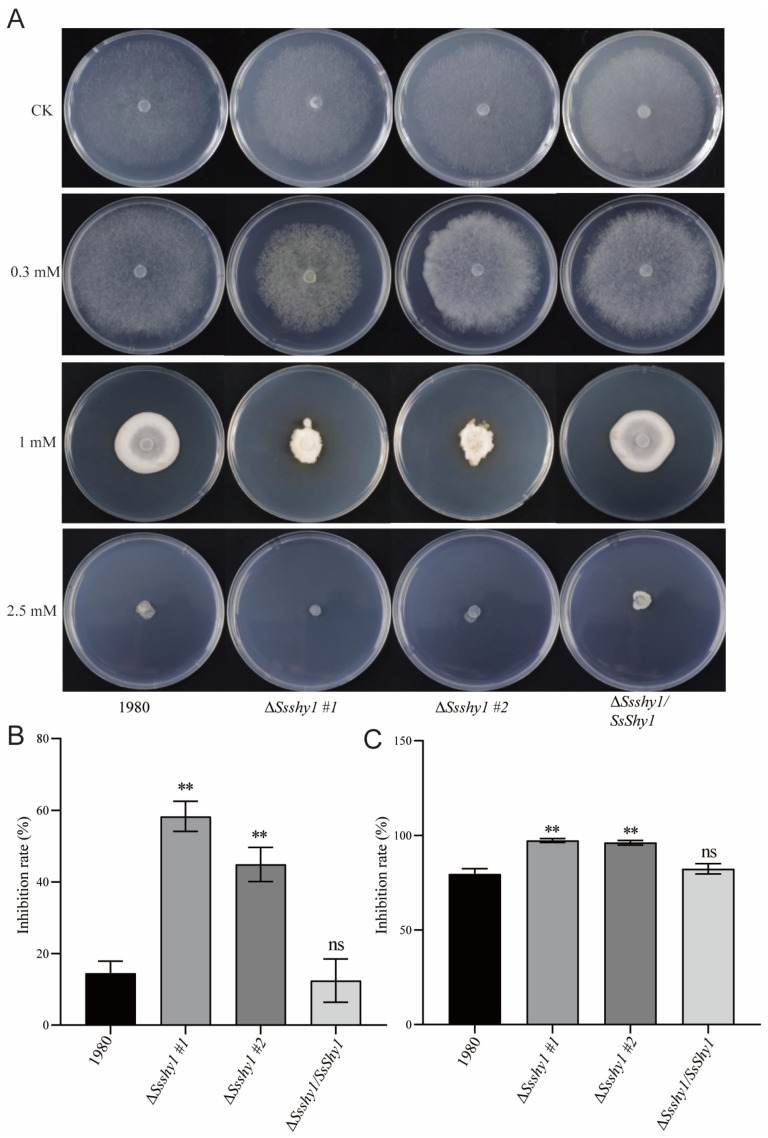
Deletion of *SsShy1* enhanced susceptibility to SA. (**A**) Growth on PDA medium containing different concentration of SA at 2 dpi. (**B**) Growth inhibition rates by 0.3 mM SA. (**C**) Growth inhibition rates by 1 mM SA. The error bars are the SDs from three independent experiments and asterisks represent significant difference between the wild type and mutants (ns, no significant difference; **, *p* < 0.01 by *t*-test).

To examine the effects of SA on the growth of *S. sclerotiorum*, Δ*Ssshy1*, Δ*Ssshy1*/*SsShy1* and the wild-type strain were cultured on PDA medium containing 0.3 mM, 1 mM, or 2.5 mM SA for 2 days. Colony diameters were measured to calculate inhibition rates. The results showed that 0.3 mM SA had little effect on the growth of the wild-type and Δ*Ssshy1/SsShy1* strains, but significantly inhibited the growth of Δ*Ssshy1* (Figure 5A,B). In comparison with the wild-type, the mycelia of Δ*Ssshy1* became dense and yellow (Figure 5A,B). When the SA concentration reached 1 mM, the mycelial growth of all strains was inhibited, the mycelia became dense, and the colony morphology turned irregular (Figure 5A). However, the Δ*Ssshy1* mutant was more severely affected than the wild-type and complemented strains, exhibiting much smaller and denser colonies (Figure 5A,C). When the SA concentration reached 2.5 mM, all strains demonstrated no growth or minimal mycelia growth at the edge of the plug (Figure 5A). These results indicated that SsShy1 plays a role in regulating the tolerance of *S. sclerotiorum* to SA.

**Figure 6:**
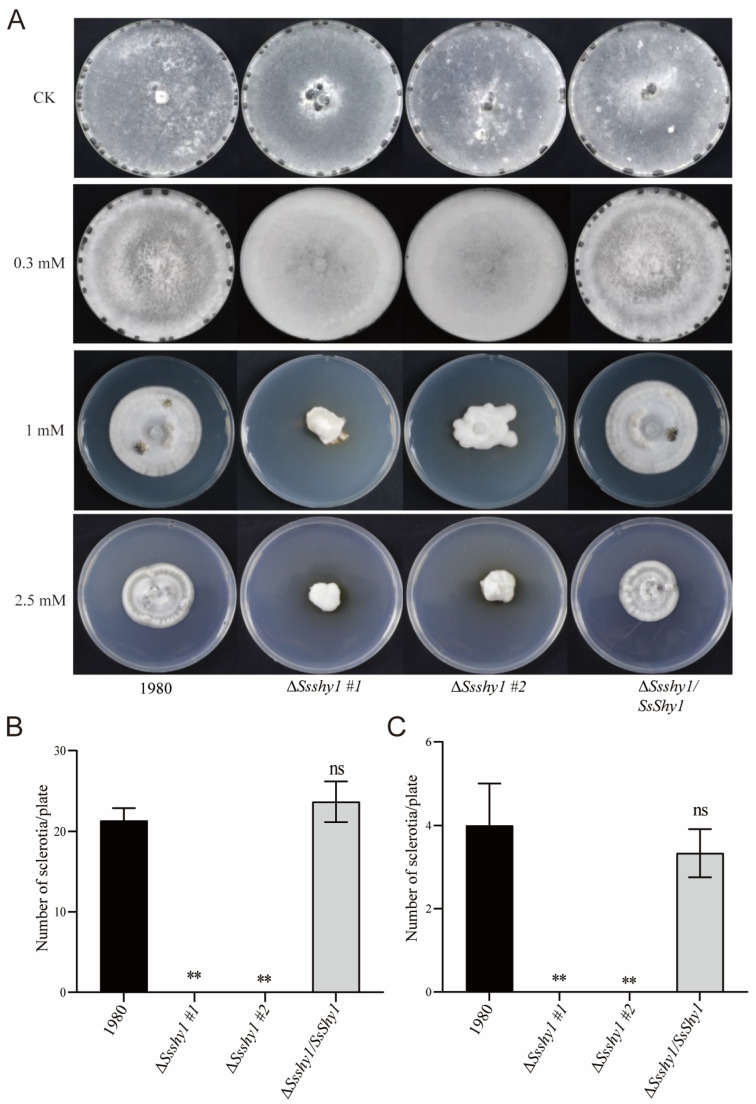
Exogenous SA affected development of sclerotia. (**A**) Sclerotia production on PDA medium containing different concentration of SA at 14 dpi. (**B**) The number of sclerotia produced on PDA medium containing 0.3 mM SA. (**C**) The number of sclerotia produced on PDA medium containing 1 mM SA. The error bars are SDs from three independent experiments and asterisks represent significant difference between the wild type and mutants (ns, no significant difference; **, *p* < 0.01 by *t*-test).

Sclerotia is a vital form in the life cycle of *S. sclerotiorum* and plays an important role in the infection cycle. To investigate the effect of exogenous SA on the formation and development of sclerotia, the Δ*Ssshy1*, Δ*Ssshy1*/*SsShy1* and wild-type strains were cultured on PDA medium containing 0.3 mM, 1 mM, or 2.5 mM SA for 14 days. The wild-type strain and Δ*Ssshy1*/*SsShy1* could produce sclerotia, but Δ*Ssshy1* could not on PDA medium containing three concentrations of SA (Figure 6A). The quantity of sclerotia produced by the wild-type strain and Δ*Ssshy1*/*SsShy1* decreased with the increase in SA concentration. (Figure 6A–C). These results indicate that SsShy1 is involved in the formation and development of sclerotia, and that SA can inhibit this process.

### 3.6. SsShy Was Required for the Virulence of S. sclerotiorum

**Figure 7:**
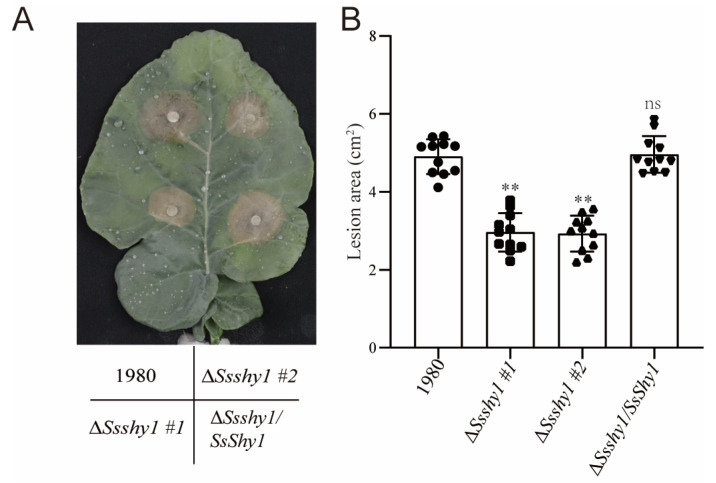
Deletion of *SsShy1* reduced the virulence of *S. sclerotiorum*. (**A**) Detached leaves of *B. napus* were inoculated with mycelium plugs of Δ*Ssshy1*, Δ*Ssshy1*/*SsShy1* and the wild type. Pictures were taken at 36 hpi. (**B**) Lesion sizes on *B. napus* leaves at 36 hpi. The black circles/squares/triangles in the column present replicates in one experiment. The error bars represent SD from 11 replicates. Asterisks represent significant differences (ns, no significant difference; **, *p* < 0.01 by *t*-test).

SA plays an important role in host resistance during the interaction between *S. sclerotiorum* and plants [24,25,26,27]. To determine whether SsShy1 is related to the virulence of *S. sclerotiorum*, we inoculated mycelium plugs of Δ*Ssshy1*, Δ*Ssshy1*/*SsShy1* and the wild type onto detached leaves of *B. napus*. All strains could infect *B. napus* leaves normally at 36 hpi; the symptoms caused by Δ*Ssshy1*/*SsShy1* were similar to those of the wild type. However, lesions caused by Δ*SsShy1* were significantly smaller than those caused by the wild type or Δ*Ssshy1*/*SsShy1*, indicating that the virulence of Δ*SsShy1* was considerably lower than that of the wild type or Δ*Ssshy1*/*SsShy1* (Figure 7). These results suggest that SsShy1 plays an essential role in pathogenicity.

## 4. Discussion

Salicylate hydroxylase plays an important role in the invasion of certain pathogenic bacteria. It has been demonstrated that numerous bacteria utilize salicylate hydroxylase to degrade plant SA and weaken host plant resistance [14,16,17]. In fungi, few genes encoding salicylate hydroxylases have been identified: one in *U. maydis* [18], *Epichloë festucae*, [31] and *Aspergillus niger* [32], two in *F. graminearum* [19,21], and one in *F. oxysporum* [22]. All of these identified salicylate hydroxylases can convert SA to catechol. In this study, we discovered numerous genes encoding salicylate hydroxylase in *S. sclerotiorum*, with some proteins located in the same clade as FgShy and FgNahG, respectively. We selected four candidates in the FgShy1 clade to assess their activity and found that only one protein exhibited salicylate hydroxylase activity. Hao et al. also discovered that one salicylate hydroxylase homolog, FgShyC, did not function as a salicylate hydroxylase [19]. In *U. maydis*, there are three salicylate hydroxylase homologs, but only Shy1 has been proven to degrade SA [18]. We have not detected other proteins in the FgShy clade or any proteins in the FgNahG clade. SsShy1, when overexpressed in *E. coli*, converted SA to catechol. Detecting SA in Δ*Ssshy1*, Δ*Ssshy1*/*SsShy1*, and the wild type, we found Δ*Ssshy1* has a much higher content of SA than Δ*Ssshy1*/*SsShy1* and the wild type (Figure S4). These results suggest that *S. sclerotiorum* can utilize SA as a carbon source to support its growth. Although the Δ*Ssshy1* mutants are impaired, they can still grow and produce sclerotia (Figure 2A), indicating that there is at least one additional salicylate hydroxylase compensating for the loss of SsShy1 function in *S. sclerotiorum*. Characterizing additional salicylate hydroxylases and constructing mutants lacking all salicylate hydroxylases will reveal the direct role of salicylic hydroxylases in growth. In the future, we will analyze the proteins in the FgNahG clade.

We also observed that the hyphae of the mutant turned yellow on plates containing SA, as previously shown for FgShy1 from *F. graminearum* [19]. The mechanism for this phenomenon is not clear.

The experimental results demonstrated that deletion of *SsShy1* did not affect colonial morphology (Figure 3A), consistent with studies on salicylate hydroxylase in smut fungus and *F. graminearum*, suggesting that salicylate hydroxylase is not involved in colonial morphology. However, deletion of *SsShy1* slowed down the mycelial growth and resulted in fewer sclerotia compared to the wild type and complementary strains (Figure 2B,C), signifying *SsShy1* as the key active enzyme in regulating the mycelial growth and development of sclerotia. The virulence of the Δ*SsShy1* mutant was considerably lower than that of the wild type (Figure 7), indicating that the absence of *SsShy1* weakened *S. sclerotiorum*’s virulence. Further analysis clarified that Δ*SsShy1* formed smaller compound appressoria in onion epidermal cells compared to the wild type and complemented strains (Figure S5). The altered mycelial growth, sclerotia development, and infection structures might be associated with the involvement of salicylate hydroxylase in the tricarboxylic acid cycle of glucose metabolism, with glucose metabolism activity being inhibited to some extent, causing nutrient deficiency during growth of the mutant.

The salicylate hydroxylase ShyA in *Aspergillus niger* exhibited activity not only towards SA but also 4-aminosalicylic acid, 2,3-dihydroxybenzoic acid, and gentisic acid [32]. The combination of ShyA and CrcA can produce *cis,cis*-muconic acid from SA. *cis*,*cis*-Muconic acid can serve as an essential platform chemical for synthesizing nylon, polyurethane, polyethylene terephthalate (PET), resins, and lubricants [33,34,35]. Whether SsShy1 can metabolize other SA-related chemicals and the extent of its salicylate hydroxylase activity require further analysis.

Earlier research has affirmed SA’s involvement in the interaction of *S. sclerotiorum* with *B. napus* [25,26] and Arabidopsis [24]. The application of exogenous SA can also enhance plant resistance to the pathogen [25]. Overexpressing *BnaNPR1* in *Nicotiana benthamiana* and *B. napus* significantly improves their resistance to *S. sclerotiorum*. Further experiments reveal that after infection with *S. sclerotiorum*, *BnaNPR1* transgenic plants activate the expression of genes related to the SA defense response [36]. Recently, Yu et al. [27] found a lesion to mimic mutant of rapeseed confers basal resistance to *S. sclerotiorum* via an SA dependent pathway. All these studies validate that SA plays an important role in resistance to *S. sclerotiorum*.

Given the crucial role of SA in plant defense, pathogens might employ salicylate hydroxylase to weaken plant defense responses and successfully infect plants [16,17]. Two bacterial salicylate hydroxylases have been identified that suppress plant immunity through SA degradation [16,17]. Among pathogenic fungi, only two salicylate hydroxylase has been found to impact pathogenicity, such as FgNahG [21] and FoSAH1 [22], indicating differences in the mechanism of salicylate hydroxylase. Here, we discovered that *SsShy1* played a significant role in the pathogenesis of *S. sclerotiorum*. It was notably up-regulated at 24 hpi in infected *B. napus* leaves (Figure 2A), implying that SsShy1 may degrade host SA and suppress SA-mediated plant defense. The absence of *SsShy1* weakened *S. sclerotiorum*’s virulence (Figure 7). SA levels in *SsShy1* transgenic plants were also lower than in wild type, making them more susceptible to *S. sclerotiorum*, similar to NahG from bacterium and FgNahG [21,37]. These findings further suggest that *SsShy1* encodes a salicylate hydroxylase and acts as a virulent factor of *S. sclerotiorum*. We further predicted the motifs of SsShy1 and found that SsShy1 has no signal peptide but rather has a transmembrane motif in the N terminal. Most part of SsShy1 including its’ FAD binding domain was outside of membrane. We speculated that SsShy1 was located in membrane and functioned outside. After transient expression in *N. benthamiana*, it was found that SsShy1 was mainly localized on the membrane (Figure S6). We are striving to experiment to check whether SsShy1 located in the membrane of *S. sclerotiorum*.

From all the results, we think SsShy1 regulates the virulence of *S. sclerotiorum* by affecting growth, development, and SA degradation. Overall, SsShy1 is the first identified salicylate hydroxylase in *S. sclerotiorum*, providing valuable research material for further exploring the direct relationship between salicylate hydroxylase and pathogen pathogenesis.

## Data Availability

All data generated or analyzed during this study are included in this article and its Supplementary Information Files.

## Supplementary Materials

The following supporting information can be downloaded at: https://www.mdpi.com/article/10.3390/jof9121169/s1, Figure S1. Validation of the *SsShy1* transgenic *A. thaliana* plants. Figure S2. Expression validation of selected candidate genes in *E. coli*. Figure S3. Construction and validation of *SsShy1* deleted mutant and complemented strain. Figure S4. SA contents in the hyphae of the Δ*Ssshy1*, Δ*Ssshy1/SsShy1* and wild-type strains. Figure S5. Compound appressoria formation on onion epidermis of the Δ*Ssshy1*, Δ*Ssshy1/SsShy1* and wild-type strains. Figure S6. Subcellular localization of SsShy1 in plant cells. *SsShy1* was cloned into plant expression vector and transiently expressed in *N. benthamiana*. The fluorescence was checked using laser scanning confocal microscope. Table S1. Primers used in this study.
