# Supplementary material for: Functional Analysis of a Salicylate Hydroxylase in Sclerotinia sclerotiorum
Source: J Fungi (Basel). 2023 Dec 5;9(12):1169. doi: 10.3390/jof9121169 (PMC10744347; doi:10.3390/jof9121169)
Supplement: Supplementary file 1 [file jof-09-01169-s001.zip › Supplemental figures legend.pdf]

Supplemental data

Figure legends

Figure S1 Validation of the *SsShy1* transgenic *A. thaliana* plants. (A) Analysis of *Bar* gene existing in the *SsShy1* transgenic *A. thaliana* plants by PCR. *AtACT2* was used as internal control. (B) Expression analysis of *SsShy1* in the *SsShy1* transgenic *A. thaliana* plants by RT-PCR. *AtACT2* was used as internal control.

Figure S2 Expression validation of selected candidate genes in *E. coli*. (A) Verification by electrophoresis in SDS-PAGE gel and staining by coomassie brilliant blue (CBB). (B) Verification by western blotting with GST antibody.

Figure S3 Construction and validation of *SsShy1* deleted mutant and complemented strain. (A) schematic diagram of *SsShy1* deleted mutant construction by splicing overlap extension (SOE)-PCR. (B) Validation of *SsShy1* deleted mutant and complemented strain by RT-PCR.

Figure S4 SA contents in the hyphae of the *ΔSsshy1*, Δ*Ssshy1*/*SsShy1* and wild-type strains.

Figure S5 Compound appressoria formation on onion epidermis of the *ΔSsshy1*, Δ*Ssshy1*/*SsShy1* and wild-type strains

Figure S6 Subcellular localization of SsShy1 in plant cells. SsShy1 was cloned into plant expression vector and transiently expressed in *N. benthamiana*. The fluorescence was checked useing laser scanning confocal microscope.
