# Supplementary material for: Functional Analysis of a Salicylate Hydroxylase in Sclerotinia sclerotiorum
Source: J Fungi (Basel). 2023 Dec 5;9(12):1169. doi: 10.3390/jof9121169 (PMC10744347; doi:10.3390/jof9121169)
Supplement: Supplementary file 1 [file jof-09-01169-s001.zip › Table S1 -.pdf]

Table S1. Primers used in this study.

Primer	Sequence (5'-3')
GST-FgShy1 F	ATCTGGTTCCGCGTGGATCCATGACACAAACATCTAGCAAGCAAAAG
GST-FgShy1 R	GTCAGTCACGATGCGGCCGCTTAAGCCAGCAACTCTCTCGCCAT
GST-02963 F	ATCTGGTTCCGCGTGGATCCATGTCAACATCCACACAACC
GST-02963 R	GTCAGTCACGATGCGGCCGCTCAAAGATTCGCCCTCCCACC
GST-12382 F	ATCTGGTTCCGCGTGGATCCATGGATAGAGCATCACACATCG
GST-12382 R	GTCAGTCACGATGCGGCCGCTTACATTTTAGCAGCATTTCCATTTTCAC
GST-04729 F	ATCTGGTTCCGCGTGGATCCATGCCCACCCAATCCGGCTT
GST-04729 R	GTCAGTCACGATGCGGCCGCTTACGCATTCATTTCTTCAATTGTCAATTTTGC
pGEX5'	GGGCTGGCAAGCCACGTTTGGTG
pGEX3'	CCGGGAGCTGCATGTGTCAGAGG
SS1G_02963 qF	TGGAAAAGGCAATTGAAGCAGT
SS1G_02963 qR	ATGCTACGACGTTCATTGTG
SS1G_12382 qF	ATATGGCGAGAGTGTTGGATAG
SS1G_12382 qR	CCAACCATGTGAGGATATCCAT
SS1G_04729 qF	CATTATCTTCGGCCAGAAACACT
SS1G_04729 qR	GCACTTTCGTCAGAAACTTTGT
tubulin-qF	TTGGATTTGCTCCTTTGACCAG
tubulin-qR	AGCGGCCATCATGTTCTTAGG
02963 UA	TCATCTCAGCCGAATACTCATT
02963 AF	CCGTTATTGAAATTGCGACCT
02963 AR	TTGACCTCCACTAGCTCCAGCCAAGCCTAGATTACTTTTGGGCGGACT
02963 OF	GAGTTGAGAAGGTAGAAGAGAGT
02963 OR	AGGACAAGTCTGCCTTTATAGT
02963 BF	AAAGGAATAGAGTAGATGCCGACCGGGTTATGGTTATGGTATGGTTGTG
02963 BR	ACAAATGACGAGCGAATTCT
H853	GACAGACGTCGCGGTGAGTT
HYGF	GGCTTGGCTGGAGCTAGTGGAGGTCAA
HYGR	AACCCGCGGTCGGCATCTACTCTATTC
YGF	GATGTAGGAGGGCGTGGATATGTCCT
HYR	GTATTGACCGATTCCTTGCGGTCCGAA
SS1G_02963-CF	AGGGAACAAAAGCTGGGTACCTAGTGGTAAGGTCTTCTCCCACTGT
SS1G_02963-CR	GCCGCCGCCGCCGCCAAGCTTAAGATTCGCCCTCCCACC
02963-104F	ggggacaagtttgtacaaaaaagcaggcttcATGTCAACATCCACACAACC
02963-104R	ggggaccactttgtacaagaaagctgggtcAAGATTCGCCCTCCCACCACG
BarF	TCGACTCTAGCGAATTCCTC
BarR	ATAGGCGTCTCGCATATCTC
