# Supplementary figures and images for: Functional Analysis of a Salicylate Hydroxylase in Sclerotinia sclerotiorum
Source: J Fungi (Basel). 2023 Dec 5;9(12):1169. doi: 10.3390/jof9121169 (PMC10744347; doi:10.3390/jof9121169)

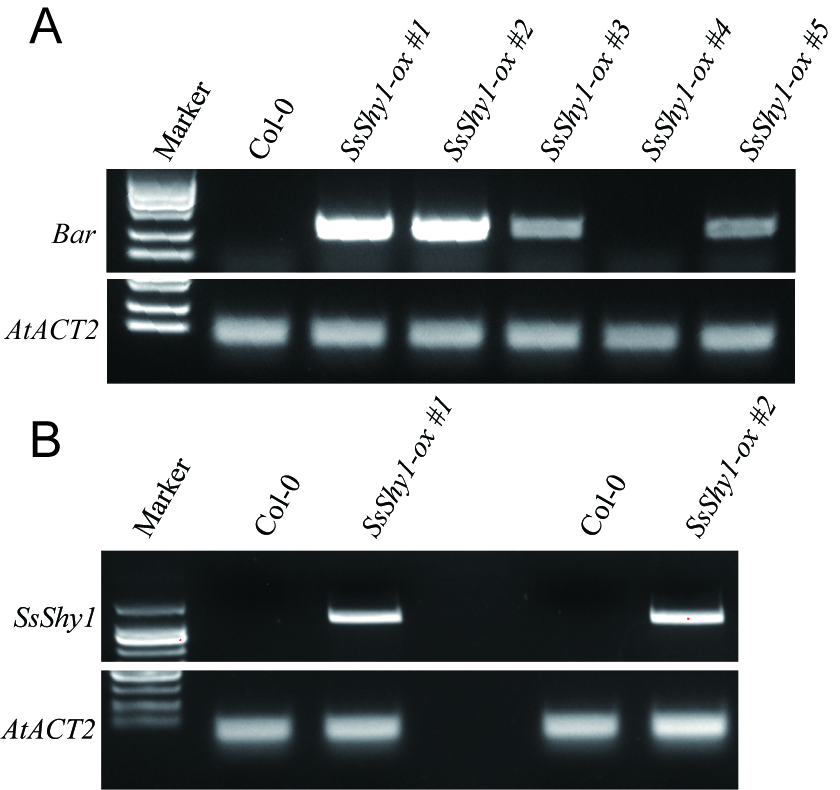

Supplement: Supplementary file 1 [file jof-09-01169-s001.zip › Figure S1.tif]

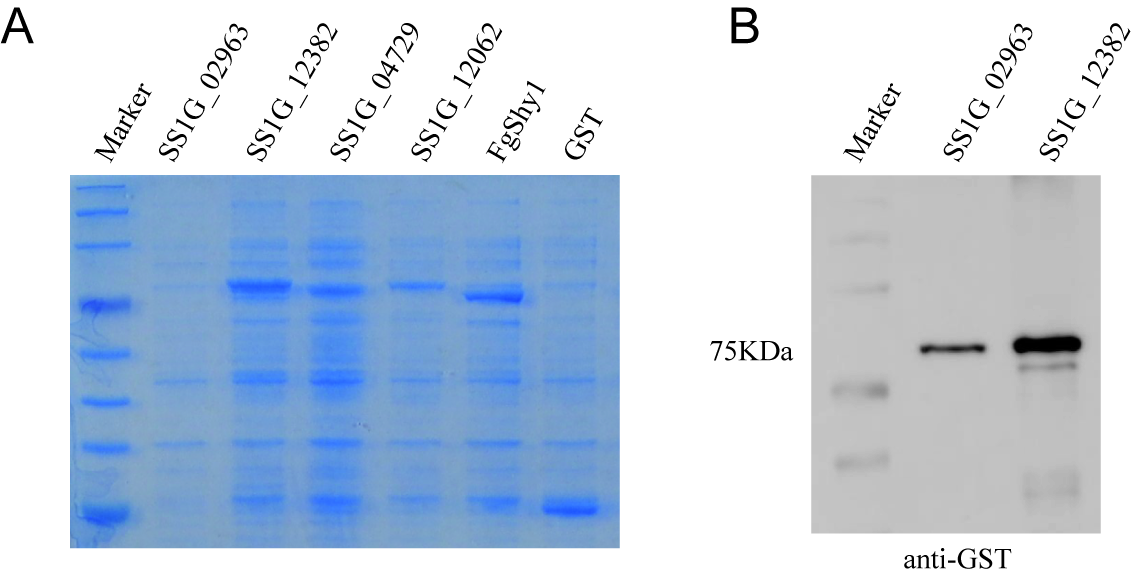

Supplement: Supplementary file 1 [file jof-09-01169-s001.zip › Figure S2.tif]

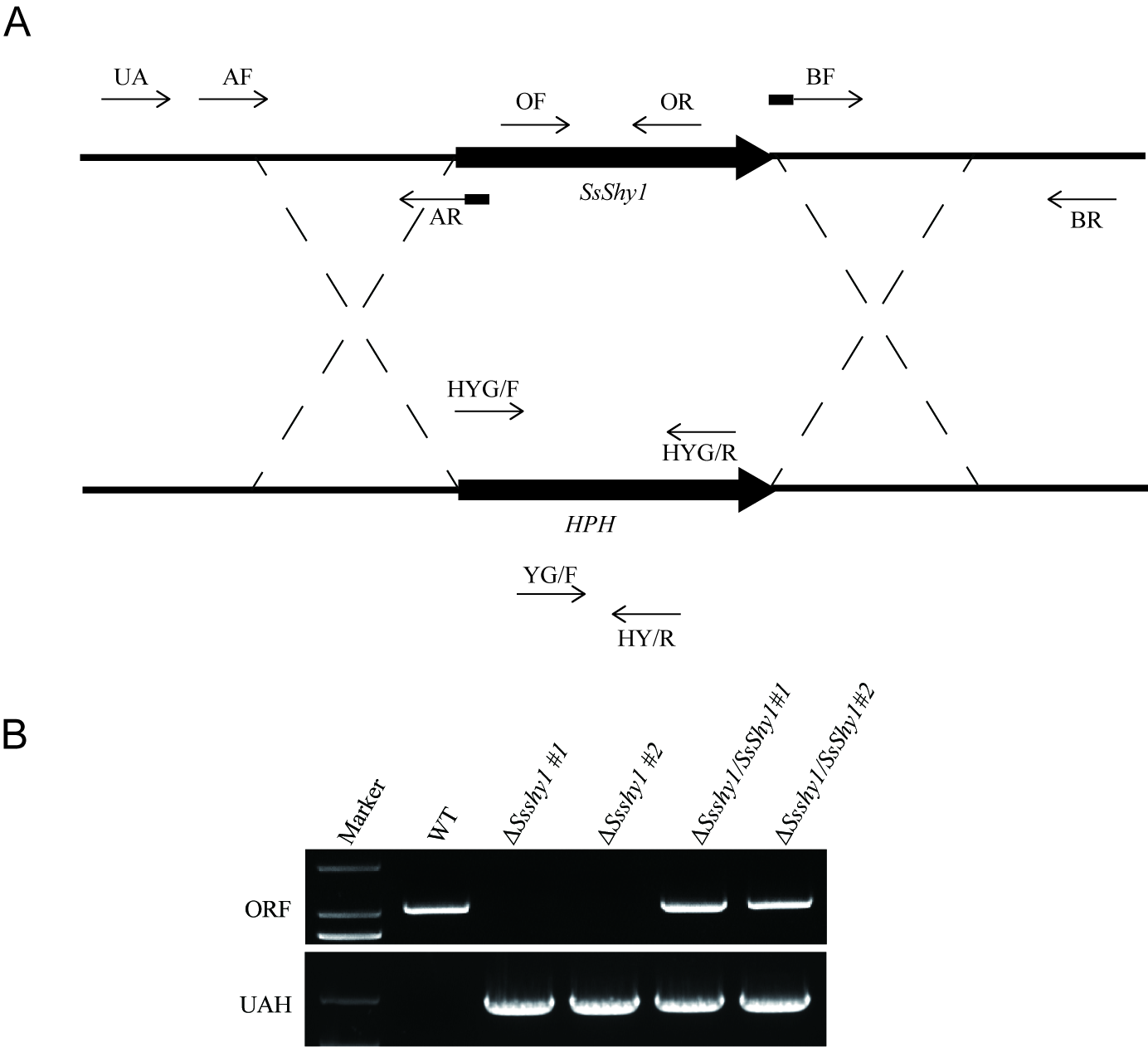

Supplement: Supplementary file 1 [file jof-09-01169-s001.zip › Figure S3.tif]

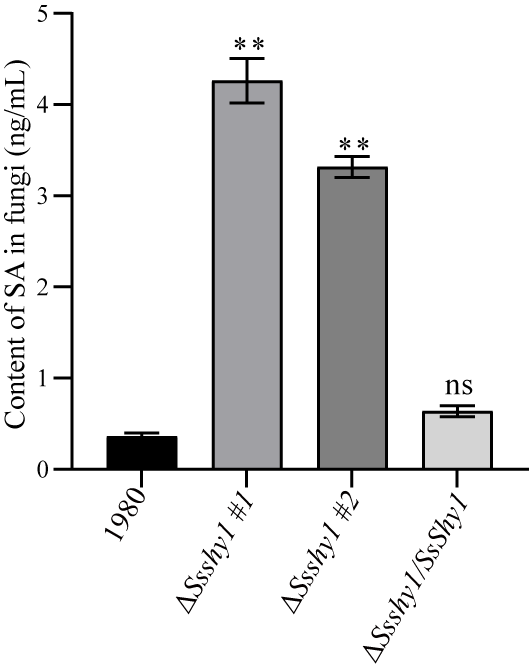

Supplement: Supplementary file 1 [file jof-09-01169-s001.zip › Figure S4.tif]

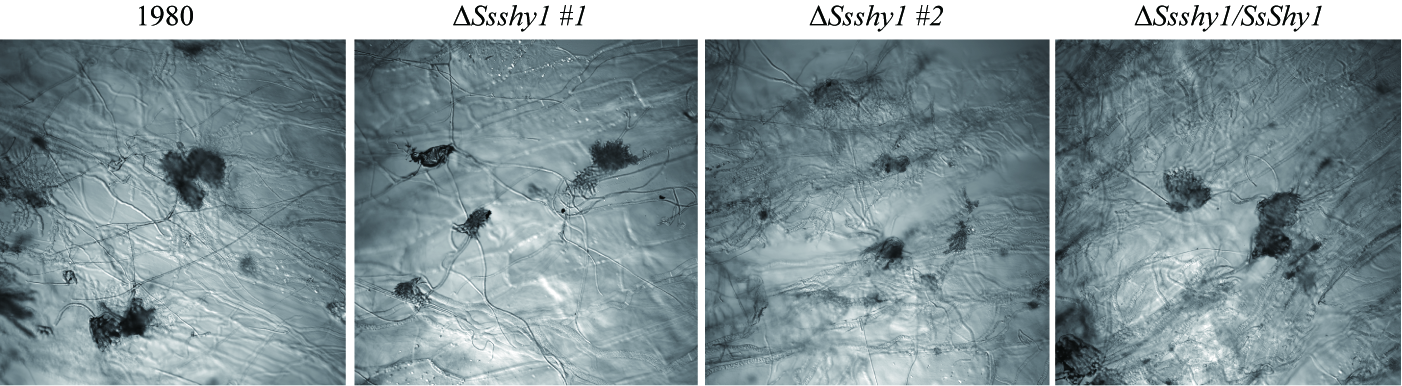

Supplement: Supplementary file 1 [file jof-09-01169-s001.zip › Figure S5.tif]

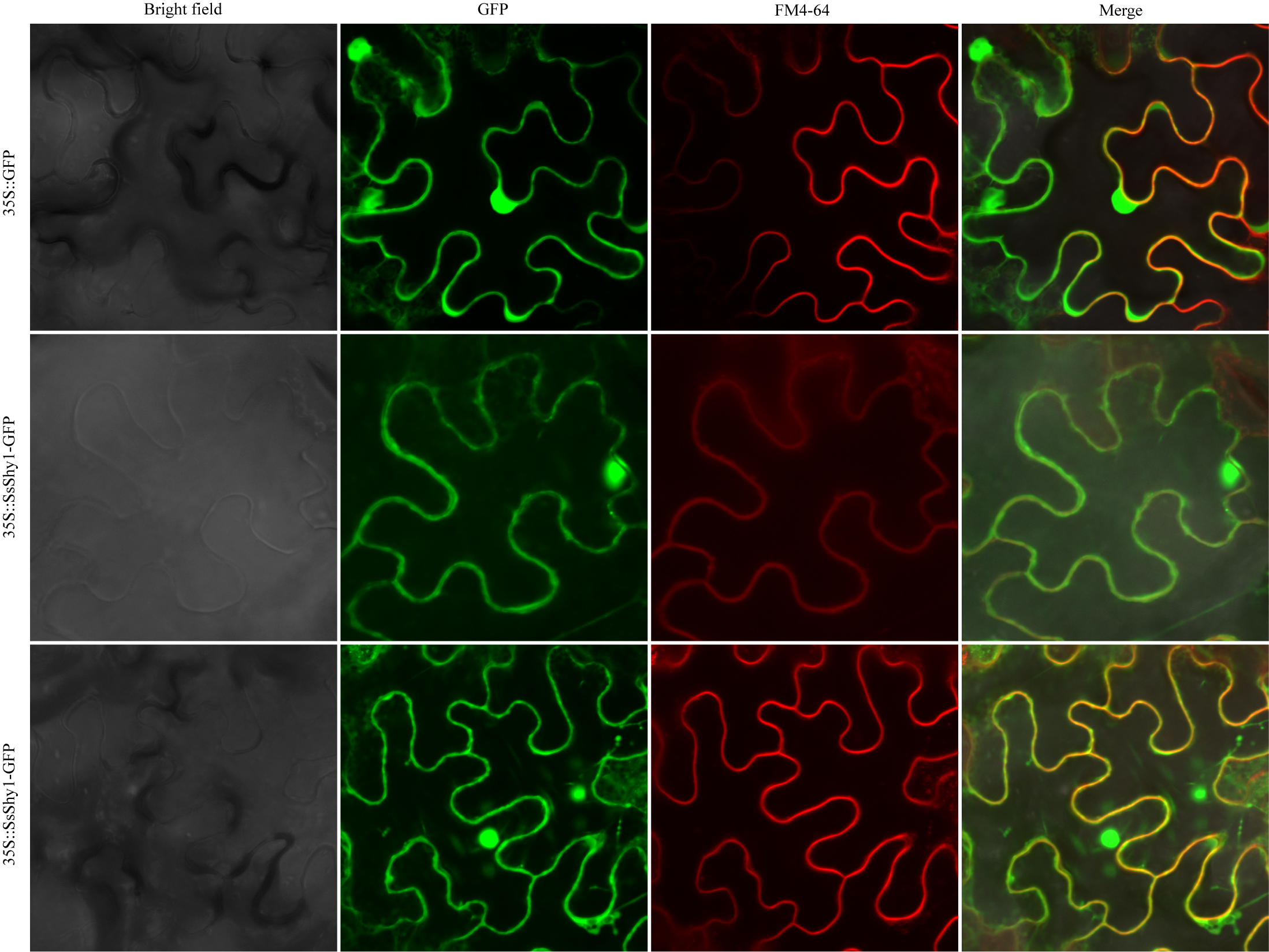

Supplement: Supplementary file 1 [file jof-09-01169-s001.zip › Figure S6.tif]
